# Supplementary material for: Nardilysin-regulated scission mechanism activates polo-like kinase 3 to suppress the development of pancreatic cancer
Source: Nat Commun. 2024 Apr 11;15:3149. doi: 10.1038/s41467-024-47242-3 (PMC11009390; doi:10.1038/s41467-024-47242-3)
Supplement: Supplementary file 1 — Supplementary Information [file 41467_2024_47242_MOESM1_ESM.pdf]

Supplementary Information

Nardilysin-Regulated Scission Mechanism Activates Polo-like Kinase 3 to Suppress the Development of Pancreatic Cancer

Jie Fu[1,10]*, Jianhua Ling[1,10], Ching-Fei Li[1], Chi-Lin Tsai[1], Wenjuan Yin[1], Junwei Hou[1], Ping Chen[1], Yu Cao[1], Ya'an Kang[2], Yichen Sun[1], Xianghou Xia[1], Zhou Jiang[1], Kenei Furukawa[1], Yu Lu[1], Min Wu[1], Qian Huang[1], Jun Yao[1], David H. Hawke[3], Bih-Fang Pan[3], Jun Zhao[4], Jiaxing Huang[1], Huamin Wang[4,5], El Mustapha Bahassi[6], Peter J. Stambrook[6, †], Peng Huang[3,7], Jason B. Fleming[2,8], Anirban Maitra[4,5], John A. Tainer[1], Mien-Chie Hung[1,9], Chunru Lin[1,5]*, and Paul J. Chiao[1,5]*

Correspondence to: jfu3@mdanderson.org; clin2@mdanderson.org; pjchiao@mdanderson.org

Supplementary Fig. 1

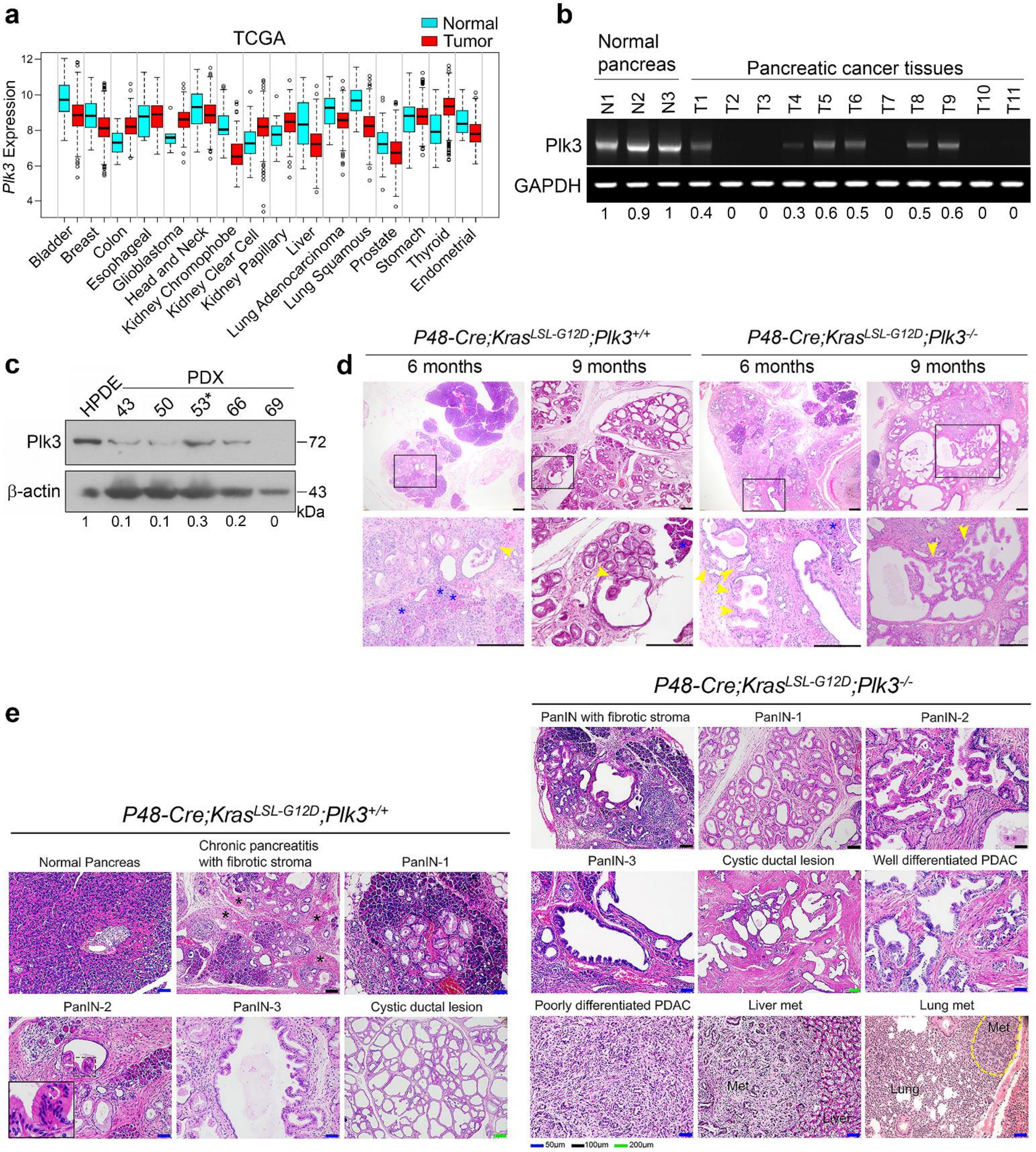

Supplementary Figure 1. Plk3 expression is reduced in PDAC and deleting *Plk3* in *p48-cre;Kras*$^{LSL-G12D}$ mice promoted PDAC and metastasis.

(**a**) RNA sequencing analysis of The Cancer Genome Atlas (TCGA) dataset [27] showing differences in *Plk3* expression between tumor and normal tissue samples. Box plots indicate minima (lower end of whisker), maxima (upper end of whisker), median (center), 25th percentile (bottom of box), and 75th percentile (top of box). (**b**) RT-PCR detection of *Plk3* mRNA expression in normal human pancreatic tissue and pancreatic tumor samples. (**c**) Immunoblot of p72Plk3 in a panel of PDX cell lines and HPDE cells. (**d**) Hematoxylin and eosin stains of PanIN lesions in pancreatic tissues, obtained from 6- and 9-month-old *p48-cre;Kras*$^{LSL-G12D}$*;Plk3*$^{+/+}$ (Plk3-WT) and *p48-cre;Kras*$^{LSL-G12D}$*;Plk3*$^{-/-}$ (Plk3-KO) mice. Squares show the magnified regions; blue asterisks indicate normal acinar tissues. Mice harboring WT Plk3 revealed abundant normal acinar tissue (>60%) at 6 months. Higher-grade PanIN lesions (PanIN-1B and PanIN-2, yellow arrowheads) showed papillary architecture and more significant loss of polarity. Compared with Plk3-WT mice, Plk3-KO mice revealed a strongly increased PanIN area and grading with large and multiple PanIN-1B or PanIN-2 lesions surrounded by an intense fibrous stroma. Scale bars, 200 µm. (**e**) Hematoxylin and eosin stains of pancreatic, liver, and lung tissues and lesions obtained from 9-month-old *p48-cre;Kras*$^{LSL-G12D}$*;Plk3*$^{+/+}$ and *p48-cre;Kras*$^{LSL-G12D}$*;Plk3*$^{-/-}$ mice. Asterisks show chronic pancreatitis with PanIN lesions and fibrotic stroma; inset shows cellular atypia and papillary architecture of PanIN-2. Scale bars: blue, 50 µm; black, 100 µm; green, 200 µm. Data in (**b, c**) are representative of two independent experiments with similar results. Source data are provided as a Source Data file.

Supplementary Fig. 2

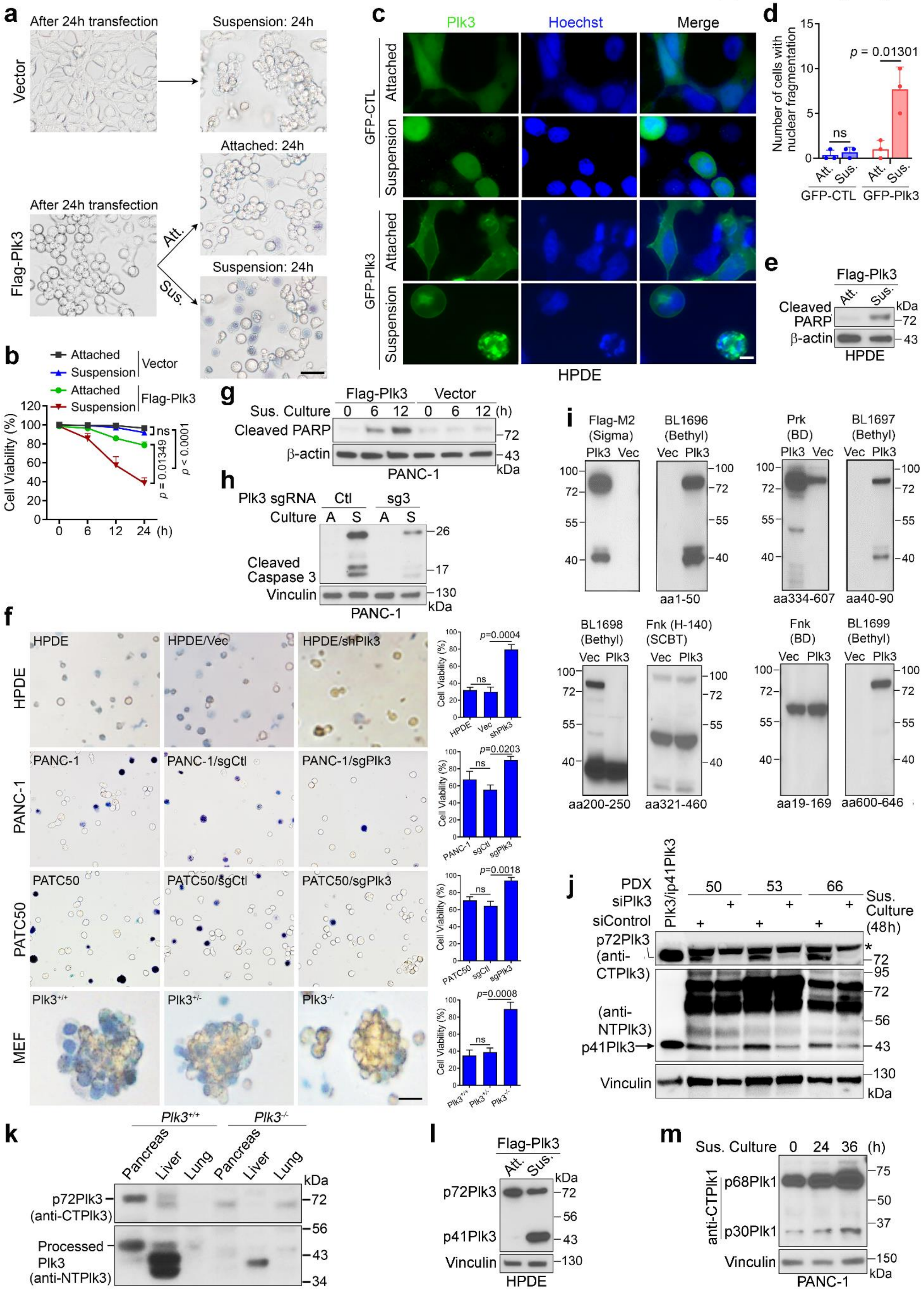

Supplementary Figure 2. p41Plk3 expression triggers anoikis.

(**a**) 293T cells were transfected with vector or Flag-Plk3. After 24 h, cells were cultured on tissue culture plates (attached) or polyHEMA-coated plates in suspension, and then stained with trypan blue. Bar, 100 µm. (**b**) Quantification of viability of cells in (**a**) for the indicated times. (**c**) Immunostaining of Plk3 in HPDE cells transfected with GFP-Plk3 and GFP-control. Hoechst: nuclear counterstaining. Bar, 10 µm. (**d**) Quantitation of cells with signs of apoptosis (fragmented nuclei). The percentage of cells showing nuclear fragmentation calculated from ten independent fields of each pool. (**e**) Immunoblot of cleaved PARP in Plk3-transfected HPDE cells cultured on tissue culture plates (Att.) or polyHEMA-coated plates in suspension (Sus.). (**f**) Representative images of trypan blue–staining cells and quantification of viable cells for HPDE cells and HPDE cells with Plk3 knockdown; PANC-1 and PATC50 cells that were lentivirally transduced to express the sgRNA targeting Plk3 or non-targeting control sgRNA; and primary MEFs isolated from $Plk3^{+/+}$, $Plk3^{+/-}$, and $Plk3^{-/-}$ mice. The cells were cultured on polyHEMA-coated plates for 36 h. Bar, 200 µm. (**g**) Immunoblot of cleaved PARP in vector- or Plk3-transfected PANC-1 cells grown in suspension culture for the indicated times. (**h**) Immunoblot of cleaved caspase-3 in Plk3 knockout PANC-1 cells grown in attached or suspension culture. (**i**) Plk3 antibody characterization. Ectopically expressed Flag-tagged Plk3 was used to characterize Plk3 antibodies from a different source. The antibody immunogens corresponding to Plk3 amino acids are shown at the bottom. (**j**) Immunoblot of Plk3 cleavage in the indicated PDAC cells transfected with Plk3 siRNAs. The first lane was p72Plk3 or p41Plk3 overexpression as a positive control. N-terminus specific anti-Plk3 antibody used for detection of p72Plk3 and p41Plk3; C-terminus specific anti-Plk3 antibody used for detection of p72Plk3 (*, unspecific band; arrow, p41Plk3). (**k**) Immunoblot of p72Plk3 and processed Plk3 in the indicated tissues from $Plk3^{+/+}$ and $Plk3^{-/-}$ mice using antibodies against N terminus or C terminus of Plk3. (**l**) Immunoblot of p72Plk3 and p41Plk3 in Plk3 transfected HPDE cells in attached and suspension culture. (**m**) Immunoblot of p68Plk1 and p30Plk1 (C-terminal region of Plk1) using C-terminus specific anti-Plk1 antibody in PANC-1 cells grown in suspension culture. In (**a, b**, **f**) the percentage of viable cells are calculated from three independent fields of each pool. Error bars, mean ± SEM (**b, d, f**), *n* = 3 independent experiments (**a-b,**

c-d, f), two-tailed unpaired t test (**b, d, f**). Data are representative of two independent experiments with similar results (**e**, **g-m**). Source data are provided as a Source Data file.

Supplementary Fig. 3

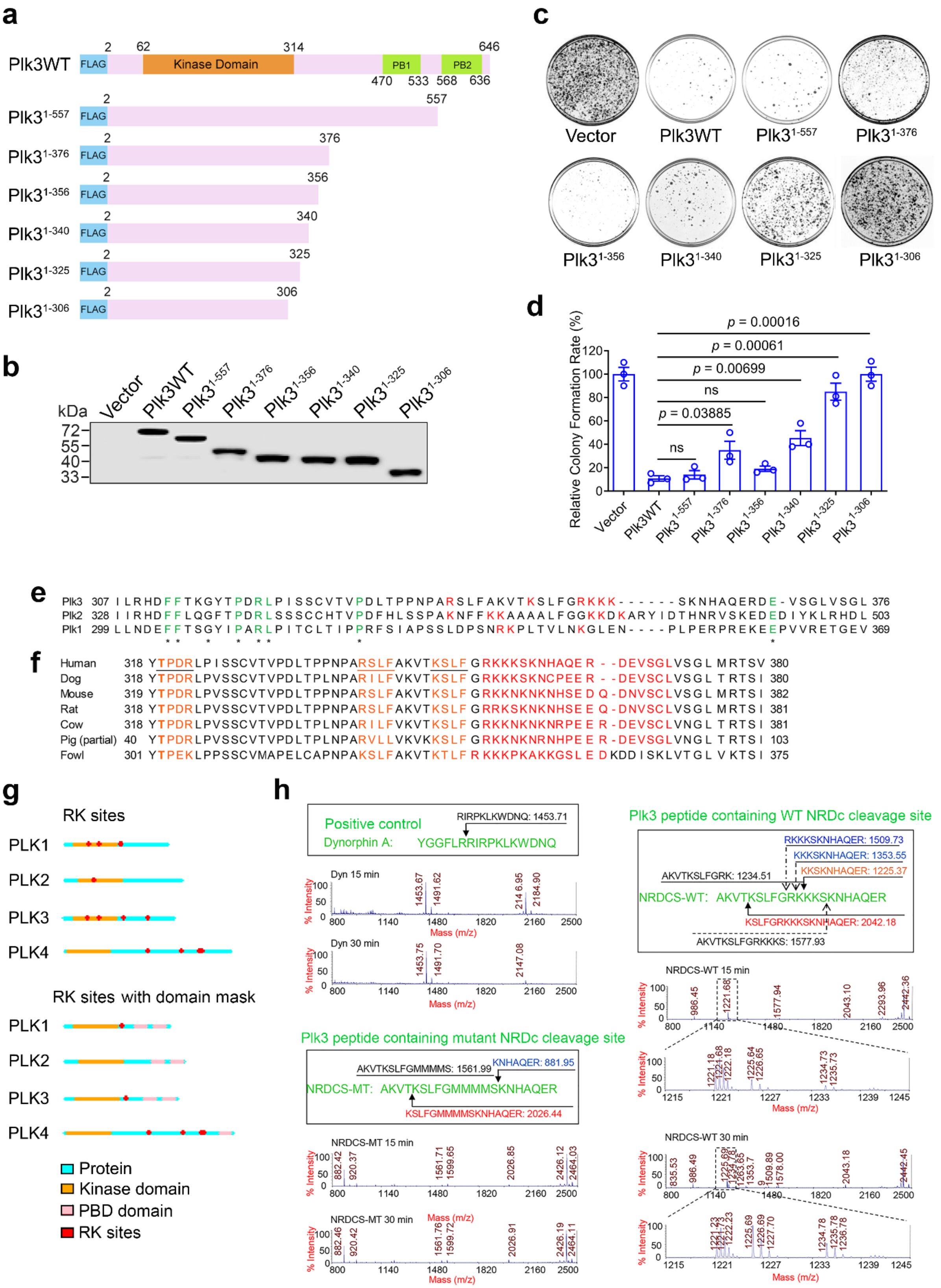

Supplementary Figure 3. p41Plk3 is generated from scission of precursor p72Plk3 at Arg354 by NRDC.

(**a**) Schematic diagram of full-length Flag-tagged p72Plk3 protein (Plk3WT) and Plk3-deletion mutants. (**b**) Immunoblot of the Plk3 expression constructs in **a**; the experiment was repeated a second time with similar results. (**c, d**) Colony formation assay from 293T cells transfected with the indicated p72Plk3WT and deletion mutants in **a**. Error bars, mean ± SEM, n = 3 independent experiments, two-tailed unpaired t test. (**e, f**) Sequence alignment of human Plk3 in Plk family members (**e**) and in Plk3 across species (**f**). (**g**) Analysis of nardilysin cleavage site (-RK-) in PLK protein family. Top, RK sites in PLK primary sequence; bottom, RK sites with domain mask. (**h**) MALDI-mass spectrometric analysis of NRDC cleavage of Plk3 peptides. The peptides include the positive control dynorphin A peptide (Dyn) with a WT NRDC cleavage site, Plk3 peptide with a WT or mutant NRDC cleavage site. The major and minor peaks in MALDI-mass spectrometric analysis and corresponding cleaved peptide sequences were identified. Source data are provided as a Source Data file.

Supplementary Fig.4

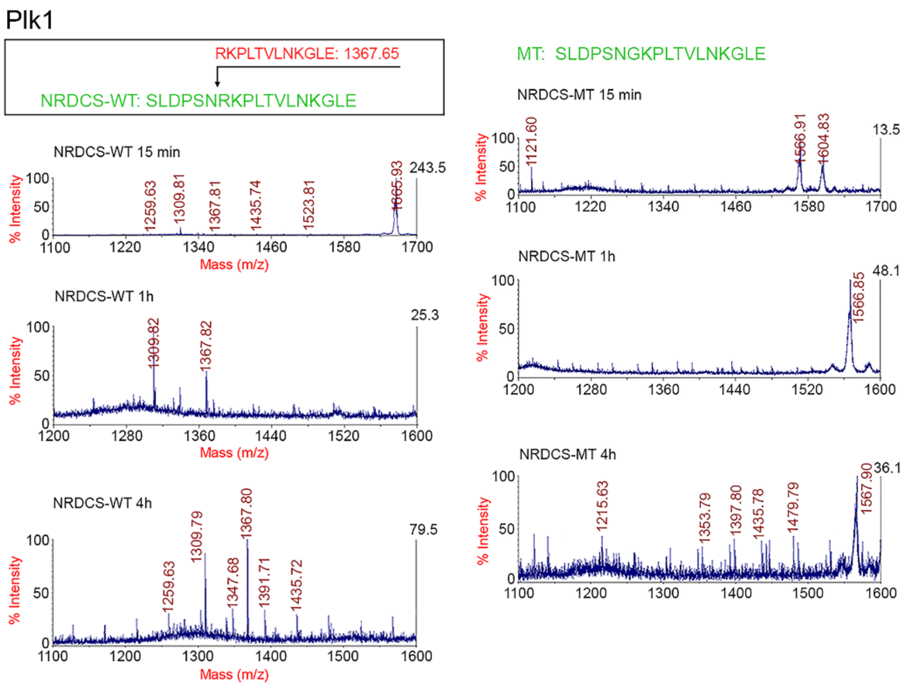

Supplementary Figure 4. MALDI-mass spectrometric analysis of NRDC cleavage of Plk1 peptides. The peptides include WT Plk1 peptide (left) and Plk1 peptide with a mutant NRDC cleavage site (right). The major and minor peaks in MALDI-mass spectrometric analysis and corresponding cleaved peptide sequences were identified.

Supplementary Fig. 5

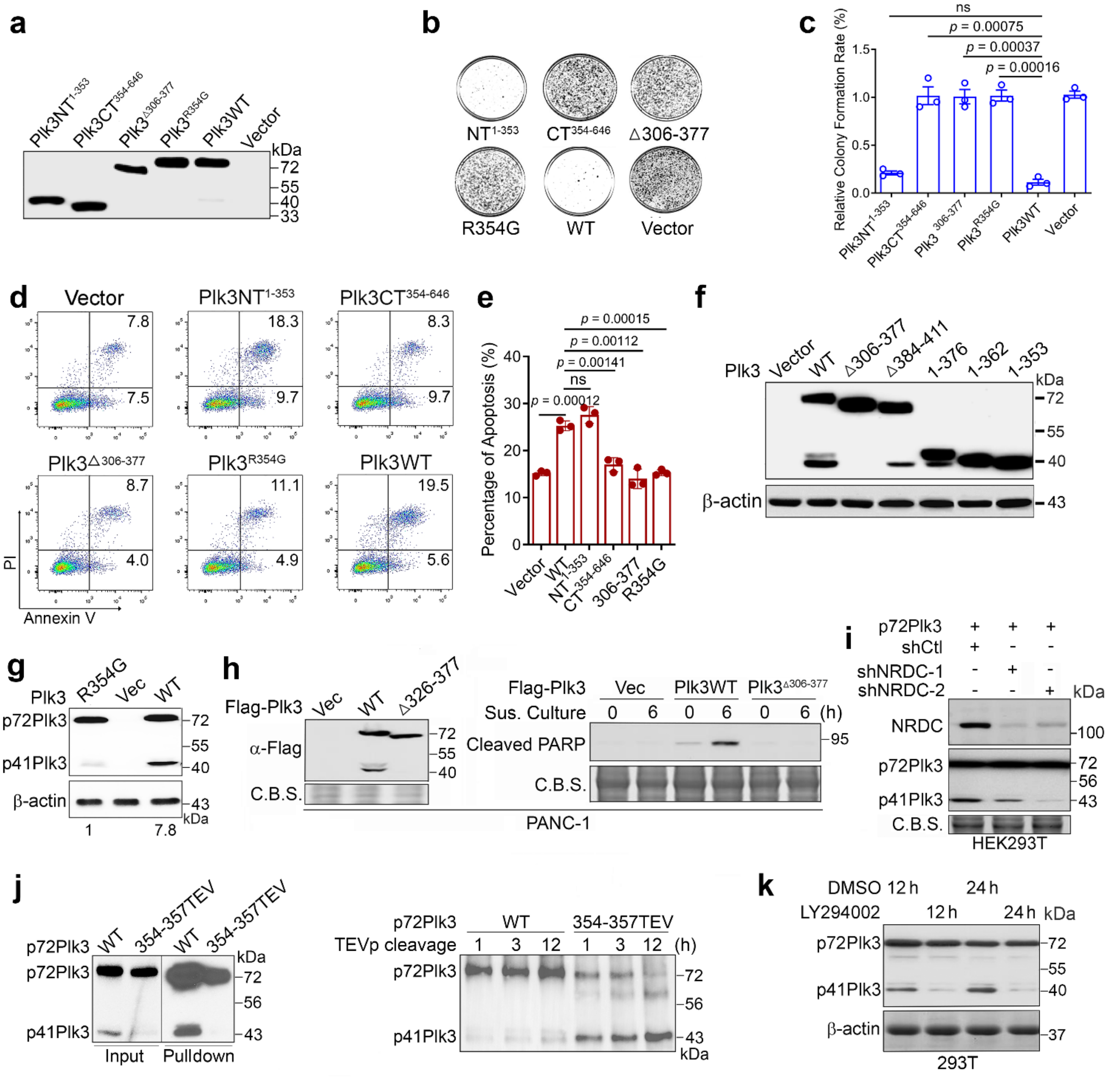

Supplementary Figure 5. p41Plk3 and p38Plk1 are generated from the scission of catalytic inactive p72Plk3 and p68Plk1 kinases, respectively.

(**a**) Immunoblot of the indicated Flag-tagged Plk3 or mutants. (**b**-**e**) Colony-formation assay (**b, c**) and flow cytometry analysis of apoptosis-inducing activity (**d, e**) from 293T cells transfected with the indicated Plk3 constructs in **a**. (**f**) Immunoblot of Plk3 in 293T cells transfected with the indicated Flag-Plk3 or mutants. (**g**) Immunoblot of the Plk3 cleavage in 293T cells transfected with the indicated Plk3WT or mutant. The p41Plk3:p72Plk3 ratios are shown at the bottom. (**h**) Immunoblots of p72Plk3 and p41Plk3 (left) and cleaved PARP (right) at the indicated times in PANC-1 cells transfected with indicated Plk3 or Plk3 mutants. (**i**) Immunoblot of p41Plk3 expression in 293T cells that were lentivirally transduced to express the shRNA targeting nardilysin, followed by overexpression of p72Plk3. (**j**) Left, immunoblot of p72Plk3 and p41Plk3 in Flag-p72Plk3–transfected cells with NRDC cleavage motif RKKK replaced with tobacco etch virus (TEV) protease cleavage sequence ENLYFQG (354-357TEV). Right, lysates from Flag-pulldown p72Plk3 WT and 354-357TEV mutant were incubated with recombinant TEV protease at the indicated time points, then immunoblotted for detection of p72Plk3 cleavage. (**k**) p72Plk3 and p41Plk3 expression in Plk3-transfected 293T cells treated with PI3K inhibitor LY294002 (20 μM) for indicated times. Data in (**a, f-k**) are representative of two independent experiments with similar results. Error bars, mean ± SEM, *n* = 3 independent experiments (**c, e**), two-tailed unpaired t test (**c, e**). Source data are provided as a Source Data file.

Supplementary Fig. 6

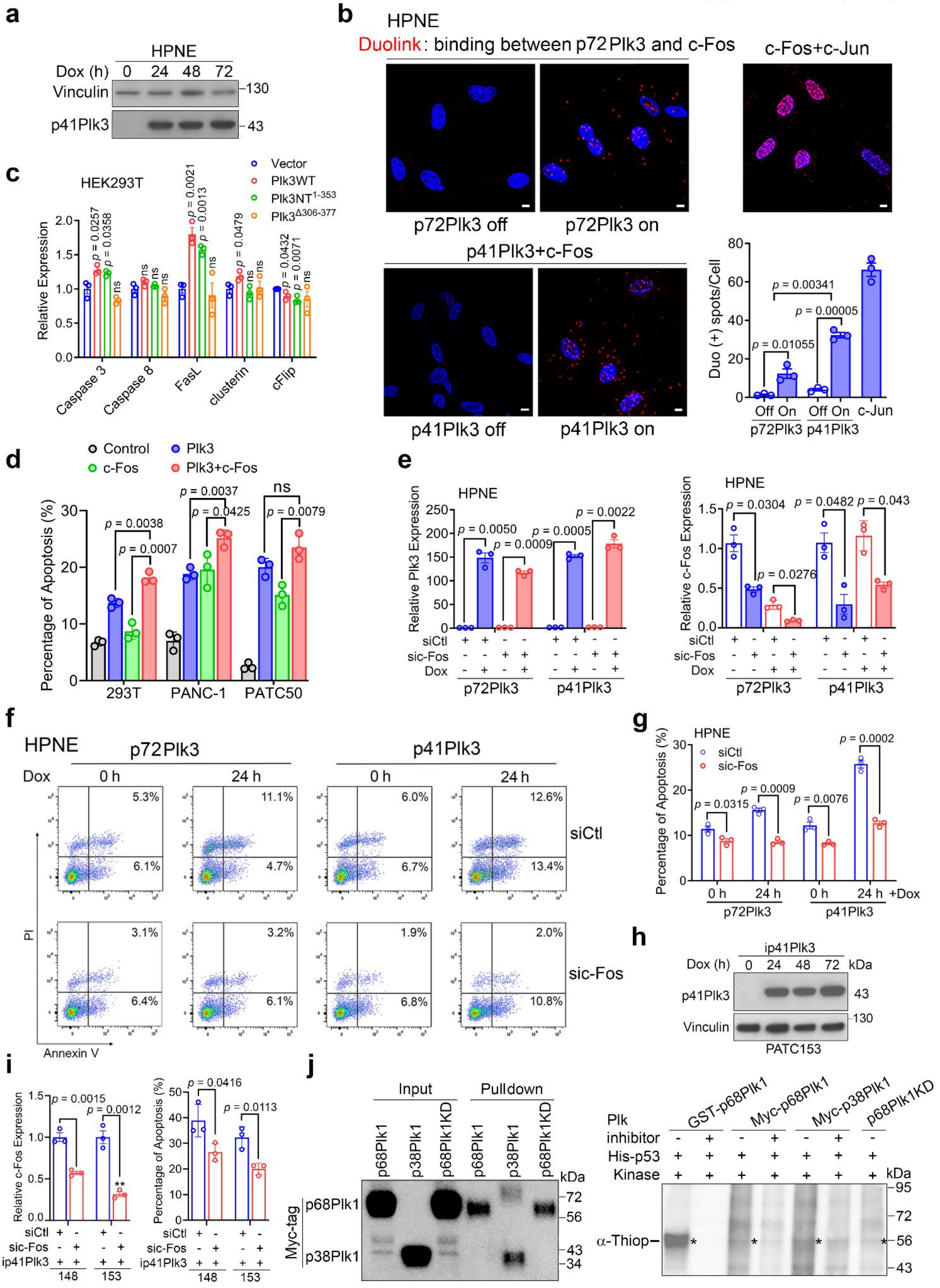

Supplementary Figure 6. Identifying feed-forward mechanisms that regulate p41Plk3-mediated apoptosis.

(**a**) Immunoblot of p41Plk3 expression under a Dox-inducible system in HPNE cells treated with Dox for the indicated times. (**b**) Results of *in situ* proximity ligation assay performed to detect interactions between p72Plk3 or p41Plk3 and c-Fos in HPNE cells with Dox-inducible expression of p72Plk3 or p41Plk3. c-Fos and c-Jun used as positive controls. Red foci indicate interactions. Number of red dots was divided by the number of nuclei. Bar, 5 µm. Bar diagram, red spots calculated from three independent fields of each pool. (**c**) qRT-PCR analysis of c-Fos–regulated pro-apoptotic and anti-apoptotic gene expression in 293T cells transfected with the indicated Plk3 constructs. FasL, Fas ligand. (**d**) 293T, PANC-1, and PATC50 cells transfected with p72Plk3, or c-Fos, or co-transfected with p72Plk3 and c-Fos were assessed for apoptosis by annexin V/PI staining followed by flow cytometry analysis. (**e**) HPNE cells with inducible p72Plk3 and p41Plk3 expression were transfected with c-Fos siRNA (sic-Fos) or control siRNA (siCtl). qRT-PCR analyses of Plk3 (left) and c-Fos (right) expression in the cells with Dox treatment for 24 h. (**f**) Representative flow cytometry plots of apoptosis-inducing activity according to annexin V/PI staining and flow cytometry as in **e**. (**g**) Quantification of apoptotic cells in **f** by flow cytometry analysis. (**h**) Immunoblot of p41Plk3 expression under a Dox-inducible system in PATC153 cells treated with Dox for the indicated times. PATC153 was derived from liver metastases of primary PDAC. (**i**) PATC148 cells or PATC153 cells with inducible p41Plk3 expression were transfected with c-Fos siRNA or control siRNA. Left, qRT-PCR detection of c-Fos expression in the cells with Dox treatment for 36 h. Right, flow cytometry analysis of apoptosis-inducing activity according to annexin V/PI staining in cells under suspension culture. (**j**) *In vitro* kinase assay using Myc-p68Plk1, Myc-p38Plk1, or Myc-p68Plk1KD immunoprecipitated from 293T cells to incubate with purified recombinant His-p53 as a substrate with or without Plk inhibitor. KD, kinase-dead (K82M) mutant; asterisk indicates p53 phosphorylation by Plk1 was detected by α-ThioP. Input and pulldown showed similar transfection and pulldown efficiency of Plk1 constructs. Data in (**a, h, j**) are representative of two independent experiments with similar results. Error bars, S.D. of three independent experiments in **b**-**g** and **i**, two-tailed unpaired t test (**b**-**g**, **i**). Source data are provided as a Source Data file.

Supplementary Fig. 7

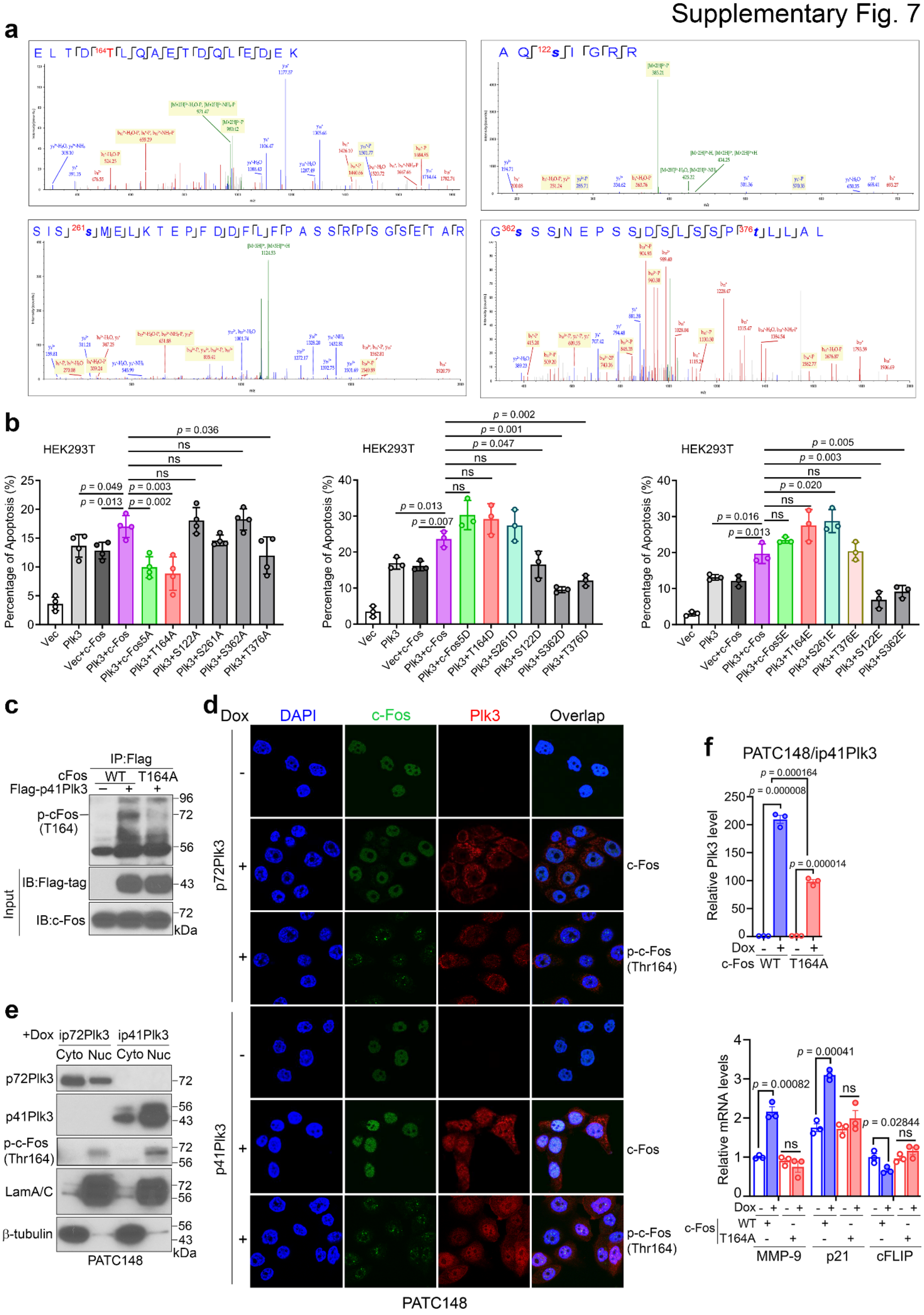

Supplementary Figure 7. Identification of Plk3 phosphorylation sites on c-Fos.

(**a**) Annotated MS/MS spectrum assigned to the c-Fos peptide sequence: ELTD[pT164]LQAETDQLEDEK, T5-Phospho (79.96633 Da), identified with: Mascot (v1.30); IonScore:59, Exp Value:8.9E-005, ions matched by search engine:11/178, fragments used for search: b; b-H_2O; b-NH_3; y; y-H_2O; y-NH_3; Sequence: AQ[pS122]IGRR, S3-Phospho (79.96633 Da), charge: +2, monoisotopic m/z:434.21347 Da (-0.01 mmu/-0.02 ppm), MH+: 867.41966 Da, RT: 9.57 min, IonScore:32, Exp Value:9.8E-003, ions matched by search engine: 5/58; Sequence: SIS[pS261]MELKTEPFDDFLFPASSRPSGSETAR, S3-Phospho (79.96633 Da), charge: +3, monoisotopic m/z:1123.85022 Da (+0.49 mmu/+0.43 ppm), MH+: 3369.53611 Da, RT: 81.56 min, IonScore:70, Exp Value:1.1E-005, ions matched by search engine: 14/330; Sequence: G[pS362]SSNEPSSDSLSSP[pT376]LLAL, S2-Phospho (79.96633 Da), T16-Phospho (79.96633 Da), charge: +2, monoisotopic m/z:1054.93677 Da (-0.53 mmu/-0.5 ppm), MH+: 2108.86626 Da, RT: 19.60 min, IonScore:37, Exp Value:1.2E-002, ions matched by search engine: 8/180. All peptides are identified with: Mascot (v1.30); Fragment match tolerance used for search: 0.8 Da; Fragments used for search: b; b-H_2O; b-NH_3; y; y-H_2O; y-NH_3. (**b**) Quantification of apoptotic cells as evaluated using annexin V/PI staining in 293T cells transfected with p72Plk3 and/or indicated c-Fos mutants. 5A, c-Fos S122A/T164A/S261A/T362A/T376A mutant; 5D, c-Fos S122D/T164D/S261D/T362D/T376D mutant; 5E, c-Fos S122E/T164E/S261E/T362E/T376E mutant. (**c**) Lysates from 293T cells co-transfected with Flag-p41Plk3 and c-Fos (WT vs T164A) were immunoprecipitated with anti-Flag and immunoblotted with anti–phospho-c-FosT164 antibody. (**d, e**) Results of immunofluorescence (**d**) and immunoblot (**e**) performed to show localization of c-Fos and phospho-c-Fos T164 and to detect interactions between p72Plk3 or p41Plk3 and c-Fos in PATC148 cells with Dox-inducible expression of p72Plk3 and p41Plk3. (**f**) qRT-PCR detection of c-Fos target genes in PATC148/ip41Plk3 cells harboring c-Fos WT or T164A mutant. Error bars, S.D. of three independent experiments in **b** and **f**, two-tailed unpaired t test (**b**, **f**). Data in (**c, e**) are representative of two independent experiments with similar results. Source data are provided as a Source Data file.

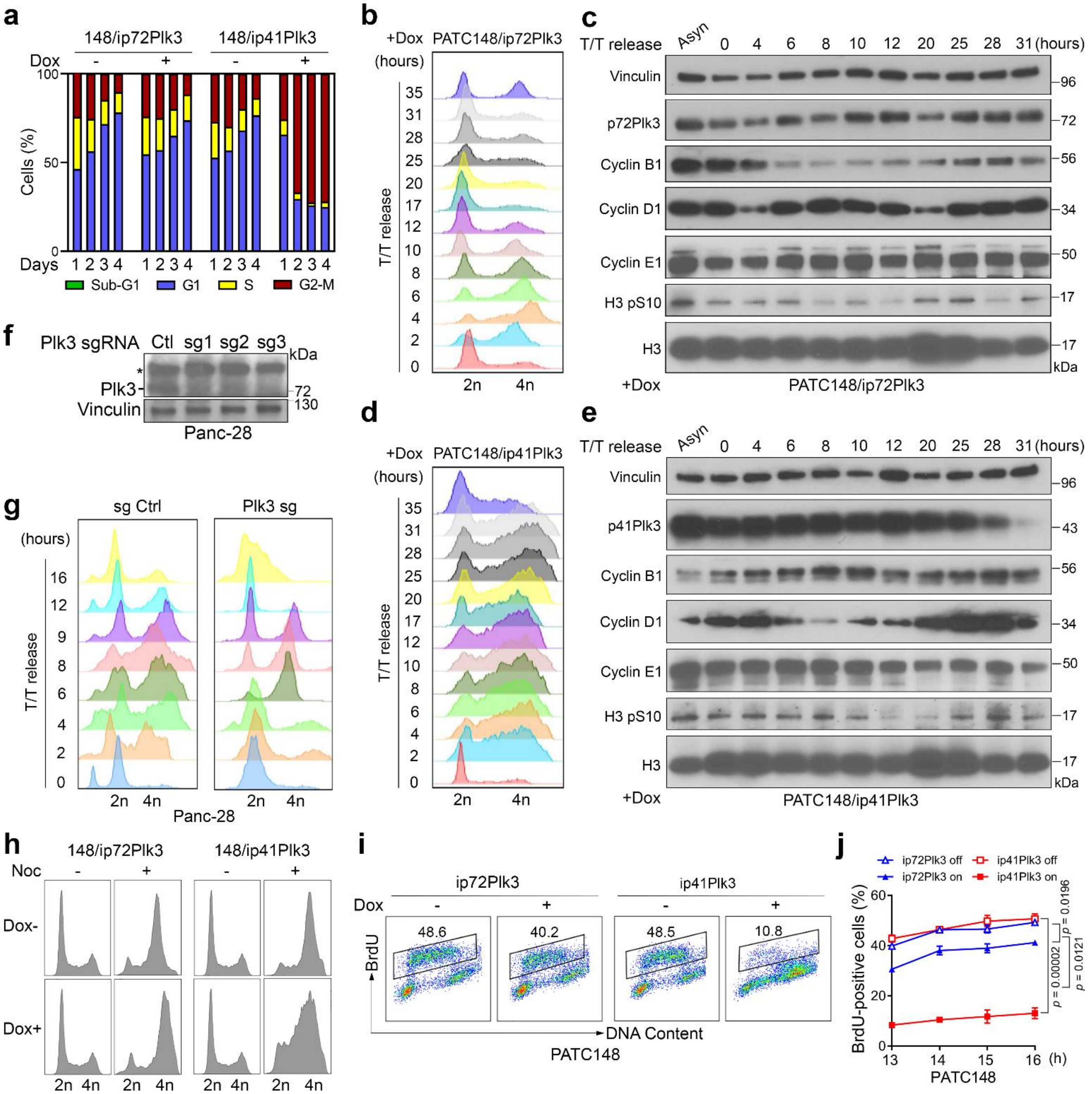
a
148/ip72Plk3
148/ip41Plk3
Dox
Cells (%)
Days
Sub-G1
G1
S
G2-M
b
+Dox
PATC148/ip72Plk3
(hours)
T/T release
2n
4n
c
T/T release
Asyn
(hours)
Vinculin
p72Plk3
Cyclin B1
Cyclin D1
Cyclin E1
H3 pS10
H3
kDa
+Dox
PATC148/ip72Plk3
d
PATC148/ip41Plk3
e
p41Plk3
PATC148/ip41Plk3
f
Plk3 sgRNA
Ctl
sg1
sg2
sg3
Plk3
Vinculin
Panc-28
g
sg Ctrl
Plk3 sg
Panc-28
h
Noc
Dox-
Dox+
i
ip72Plk3
ip41Plk3
BrdU
DNA Content
PATC148
48.6
40.2
48.5
10.8
j
ip72Plk3 off
ip41Plk3 off
ip72Plk3 on
ip41Plk3 on
BrdU-positive cells (%)
p = 0.0196
p = 0.00002
p = 0.0121
(h)
PATC148

Supplementary Figure 8. p41Plk3 plays an essential role in regulation of the G2/M phase of the cell cycle.

(**a**) The DNA content in PATC148 cells expressing an inducible p72Plk3 or p41Plk3 was determined by flow cytometry at the indicated days. (**b, d**) PATC148 cells stably transfected with an inducible p72Plk3 (**b**) or p41Plk3 (**d**) were released from thymidine block for the indicated times before analysis of DNA content by flow cytometry. (**c, e**) PATC148/ip72Plk3 and PATC148/ip41Plk3 cells treated as in **b** and **d** were subjected to immunoblotting for the indicated proteins. (**f, g**) Panc-28 cells stably transfected with sgRNA targeting Plk3 or non-targeting control sgRNA (**f**) were released from thymidine block for the indicated times before analysis of DNA content by flow cytometry (**g**). Asterisk in **f** indicates a non-specific band. (**h**-**j**) PATC148/ip72Plk3 and PATC148/ip41Plk3 cells were synchronized by growth in nocodazole (400 ng/ml) for 24 h (**h**), released for 9 h, pulsed with BrdU for 30 min, and subjected to flow cytometric analysis. Representative cell cycle profiles and quantifications of BrdU-pulse-labeled cells are shown in **i** and **j**. Error bars, mean ± SEM, $n = 3$ independent experiments, two-tailed unpaired t test. Data in (**c, e, f**) are representative of two independent experiments with similar results. Source data are provided as a Source Data file.

Supplementary Fig. 9

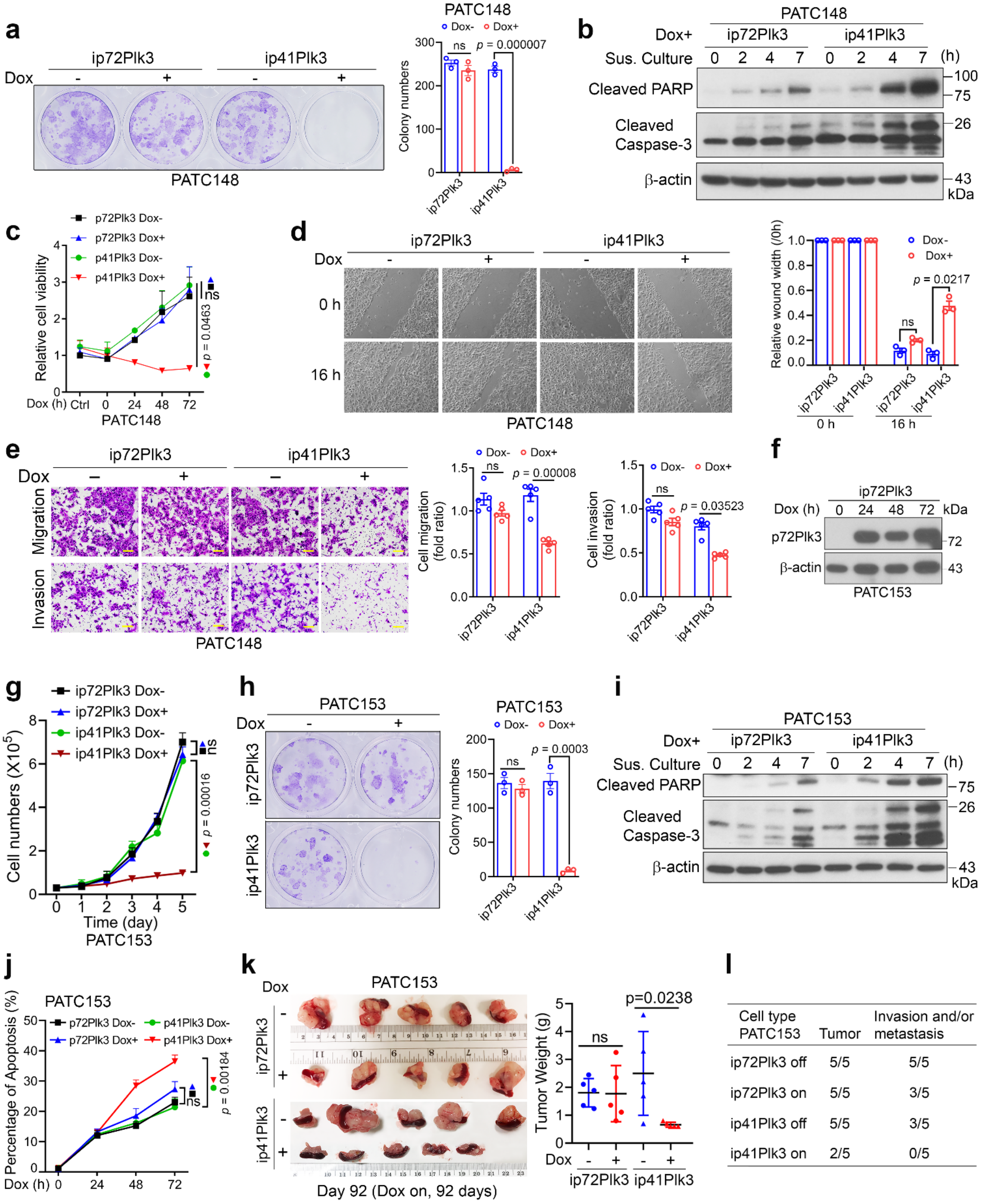

Cell type PATC153	Tumor	Invasion and/or metastasis
ip72Plk3 off	5/5	5/5
ip72Plk3 on	5/5	3/5
ip41Plk3 off	5/5	3/5
ip41Plk3 on	2/5	0/5

Supplementary Figure 9. NRDC cleavage-activated p41Plk3 inhibits migration, invasion, and PDAC progression and metastasis.

(**a**-**e**) Colony formation assay (**a**), immunoblot of cleaved PARP and cleaved caspase-3 (**b**), MTT viability assay (**c**) (*n* = 2 independent experiments), wound healing assay (**d**), and cell migration/Matrigel cell invasion assays (**e**) of PATC148 cells with Dox-inducible expression of p72Plk3 and p41Plk3. (**f**) Immunoblot of p72Plk3 expression under a Dox-inducible system in PATC153 cells treated with Dox for the indicated times. (**g**-**j**) Growth curve (**g**), colony formation assay (**h**), immunoblot of cleaved PARP and cleaved caspase-3 (**i**), and flow cytometry analysis for apoptosis-inducing activity (**j**) of PATC153 cells with inducible p72Plk3 and p41Plk3 expression. (**k**) Left, pancreatic tissues or tumors removed on day 92 from nude mice (n=5) orthotopically injected with PATC153 cells (2.5×10^5 cells) with Dox-inducible expression of p72Plk3 and p41Plk3. +, On: mice were fed with Dox-containing water upon cell inoculation and continued the treatment for the indicated times. -, off: mice were maintained Dox-free. Right, tumor weight analysis of the indicated groups in left panel. Lines represent mean ± SD (Student unpaired *t*-test). (**l**) The rates of tumor formation and metastasis of the indicated groups in **k**. Error bars represent mean ± SEM (**a, c-e, g, h, j**), *n* = 3 independent experiments (**a, d, e, g, h, j**), two-tailed unpaired t test (**a, c-e, g, h, j**). Each experiment was repeated a second time with similar results (**b**, **f**, **i**). Source data are provided as a Source Data file.

Supplementary Fig. 10

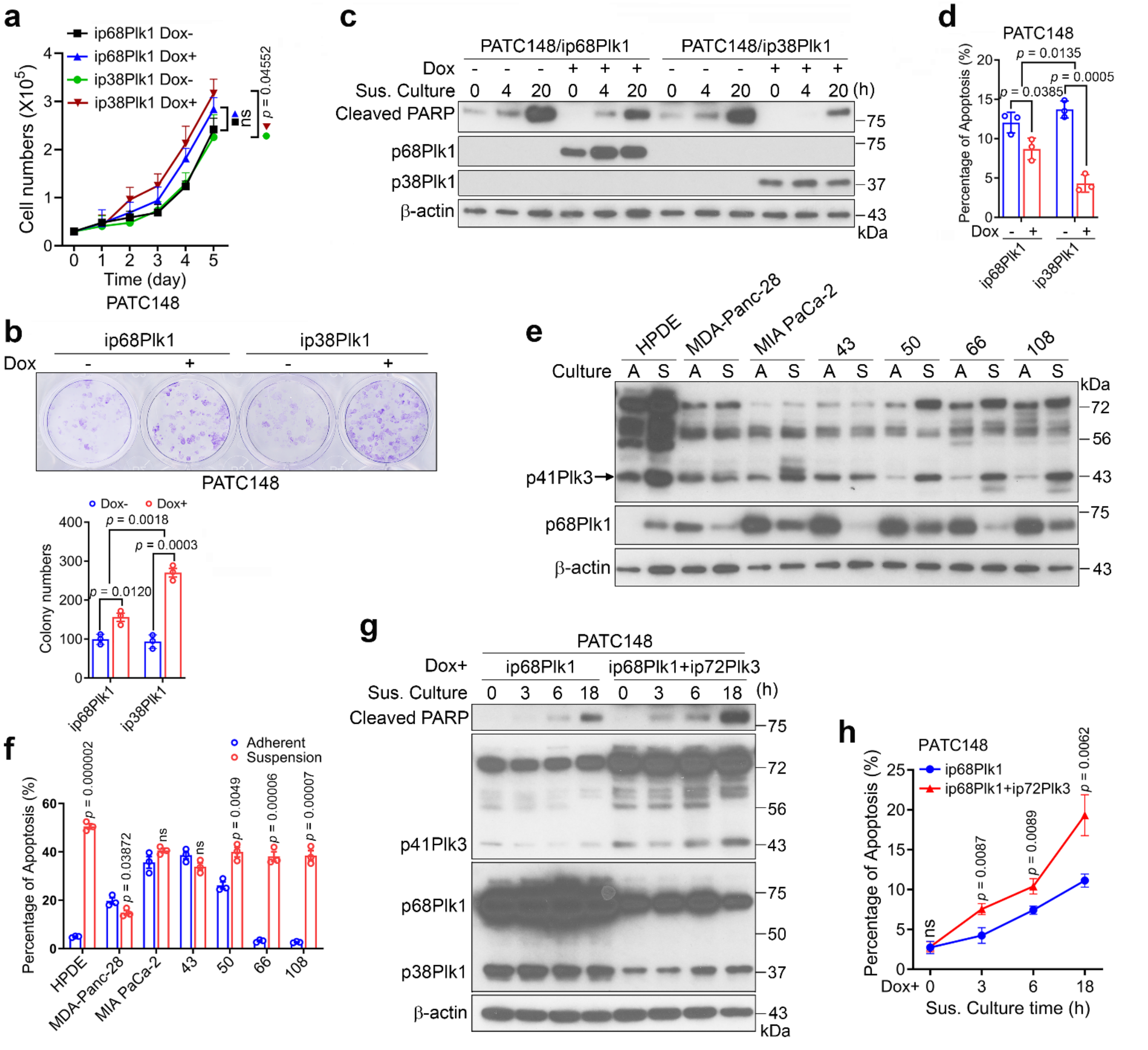

Supplementary Figure 10. NRDC proteolytic cleavage generates p41Plk3 with pro-apoptotic activity and p38Plk1 with pro-survival function, respectively.

(**a**-**d**) Growth curve (**a**), colony formation assay (**b**), immunoblot of cleaved PARP (**c**), and flow cytometry analysis of apoptosis-inducing activity (**d**) in PATC148 cells stably expressing Dox-inducible p68Plk1 or p38Plk1. (**e, f**) Immunoblot of p41Plk3 and p68Plk1 (**e**), and flow cytometry analysis of apoptosis-inducing activity (**f**) in HPDE and a panel of PDAC cells grown on adhesive (A) or polyHEMA-coated (S) plates. (**g, h**) Immunoblot of cleaved PARP, cleavage in p72Plk3 and p68Plk1 (**g**), and flow cytometry analysis of apoptosis-inducing activity (**h**) in PATC148 cells stably expressing Dox-inducible p68Plk1 or co-expressing Dox-inducible p68Plk1 and p72Plk3. Cells were grown under suspension culture conditions. Error bars, mean ± SEM, n = 3 independent experiments (**a, b, d, f, h**), two-tailed unpaired t test (**a, b, d, f, h**). Data in (**c, e, g**) are representative of two independent experiments with similar results. Source data are provided as a Source Data file.

Supplementary Table 1. cDNAs from surviving colonies that are resistant to Plk3 overexpression–induced apoptosis.

<table>
<tr><th>Clone</th><th>Gene</th><th>Accession #</th><th>Survival</th><th>Sequence</th></tr>
<tr><td>FC6-1</td><td>chorionic somatomammotropin hormone 2(CSH2)</td><td>gi|20819960</td><td>Yes</td><td>...DGSRRTGQILKQTYSKFDTNSHNHDALLKNYGLLYCFRKDMD...</td></tr>
<tr><td>SC-7-RetroN</td><td>chorionic somatomammotropin hormone 2(CSH2)</td><td>gi|20819978</td><td>Yes</td><td>...DGSRRTGQILKQTYSKFDTNSHNHDALLKNYGLLYCFRKDMDK...</td></tr>
<tr><td>C9-RetroN</td><td>corticotropinn releasing hormone(CRH)</td><td>gi|4503040</td><td>Yes</td><td>...RERRSEEPPISLDLTFHLLREVLEMARAEQLAQQAHSNRKLMEIIGK...</td></tr>
<tr><td>C21-RetroN</td><td>corticotropinn releasing hormone(CRH)</td><td>gi|4503040</td><td>Yes</td><td>...RERRSEEPPISLDLTFHLLREVLEMARAEQLAQQAHSNRKLMEIIGK...</td></tr>
<tr><td>FC-38-RetroN</td><td>annexin A1(ANXA1)</td><td>gi|4502100</td><td>Yes</td><td>...MNKVLDLELKGDIEK...MKGVGTRHKALIRIMVSR...CQAILDETKGDYEKILVALCGG..</td></tr>
<tr><td>SC-3-Retro</td><td>nbosomal protein L13a</td><td>gi|38197177</td><td>Yes</td><td>...HKTKRGQA...YDKKKRM...LKPTRKFAYL...EEKRKEKAKIHYRKKKQLMRLRKQAE...</td></tr>
</table>

Supplementary Table 2. Nardilysin cleavage in proteins

Nardilysin cleavage (-X-Arg-Lys- motif)	Proteins (n)	Largest Fragment	
		> 100 aa	< 100 aa
No site	5833		
1-3 sites	10947	10181	766
> 3 sites	3637	3564	73

p < 0.0001

NOTE: 20417 proteins were analyzed for NRDC cleavage based on sequences that contain NRDC hydrolysis motif -X-Arg-Lys- (X is not Arg or Lys). The largest proteolytic fragments are analyzed. A one-sided proportion test showed proteins containing NRDC cleavage sites (>1) were significantly more frequent than those with no cleavage sites ($p < 0.0001$). Among the proteolytic fragments or domains containing1-3 sites or >3 sites of cleavage, those of >100 aa were significantly more frequent than those of <100 aa ($p < 0.0001$, based on one-sided Fisher exact test).

Supplementary Table 3. Primers used in qRT-PCR for analysis of expression of proapoptotic genes regulated by Plk3-activated c-Fos.

Gene name	Forward Primer (5' to 3')	Reverse Primer (5' to 3')
**Plk3**	GCGCGAGAAGATCCTAAATG	GATCTGCCGCAGGTAGTAGC
**Cluster**	GTCAACGGGGTGAAACAGAT	TCAGGCAGGGCTTACACTCT
**c-Fos**	CCGGGGATAGCCTCTCTTACT	CCAGGTCCGTGCAGAAGTC
**Jagged1**	GTCCATGCAGAACGTGAACG	GCGGGACTGATACTCCTTGA
**CyclinD1**	GCTGCGAAGTGGAAACCATC	CCTCCTTCTGCACACATTTGAA
**TP53**	CAGCACATGACGGAGGTTGT	TCATCCAAATACTCCACACGC
**p21**	TGTCCGTCAGAACCCATGC	AAAGTCGAAGTTCCATCGCTC
**Bcl2**	GGTGGGGTCATGTGTGTGG	CGGTTCAGGTACTCAGTCATCC
**HB-EGF**	ATCGTGGGGCTTCTCATGTTT	TTAGTCATGCCCAACTTCACTTT
**Fas**	TCTGGTTCTTACGTCTGTTGC	CTGTGCAGTCCCTAGCTTTCC
**Bcl3**	CCGGAGGCGCTTTACTACC	TAGGGGTGTAGGCAGGTTCAC
**MMP2**	TACAGGATCATTGGCTACACACC	GGTCACATCGCTCCAGACT
**Caspase-8**	GTTGTGTGGGGTAATGACAATCT	TCAAAGGTCGTGGTCAAAGCC
**Caspase-3**	CATGGAAGCGAATCAATGGACT	CTGTACCAGACCGAGATGTCA
**cJun**	TCCAAGTGCCGAAAAAGGAAG	CGAGTTCTGAGCTTTCAAGGT
**FADD**	GCTGGCTCGTCAGCTCAAA	ACTGTTGCGTTCTCCTTCTCT
**VEGF**	AGGGCAGAATCATCACGAAGT	AGGGTCTCGATTGGATGGCA
**FBXL10**	ACAACGTCATCAGCCTAGAGTTCA GCCACA	TGGTGGGTGAAGTGGAGCCATC GGT
**Smad3**	TGGACGCAGGTTCTCCAAAC	CCGGCTCGCAGTAGGTAAC
**FasL**	CTCCGAGAGTCTACCAGCCA	TGGACTTGCCTGTTAAATGGG
**MMP9**	AGACCTGGGCAGATTCCAAAC	CGGCAAGTCTTCCGAGTAGT
**p16INK**	GATCCAGGTGGGTAGAAGGTC	CCCCTGCAAACTTCGTCCT
**cFlip**	GTCTGCTGAAGTCATCCATCAG	CTTATGTGTAGGAGAGGATAAG
**Tnfrsf10b**	ATGGAACAACGGGGACAGAAC	CTGCTGGGGAGCTAGGTCT
**PEG3**	CCTACCCAAGCACCAGTCG	GGAACTGCGTGACACATCCT
**E-cadherin**	GAACGCATTGCCACATACAC	GAATTCGGGCTTGTTGTCAT
**Vimentin**	CGAAAACACCCTGCAATCTT	TCCTGGATTTCCTCTTCGTG
