# Supplementary material for: Nardilysin-regulated scission mechanism activates polo-like kinase 3 to suppress the development of pancreatic cancer
Source: Nat Commun. 2024 Apr 11;15:3149. doi: 10.1038/s41467-024-47242-3 (PMC11009390; doi:10.1038/s41467-024-47242-3)
Supplement: Supplementary file 3 — Reporting Summary [file 41467_2024_47242_MOESM3_ESM.pdf]

nature portfolio

Corresponding author(s): Jie Fu, Chunru Lin, Paul Chiao

Last updated by author(s): Mar 5, 2024

Reporting Summary

Nature Portfolio wishes to improve the reproducibility of the work that we publish. This form provides structure for consistency and transparency in reporting. For further information on Nature Portfolio policies, see our Editorial Policies and the Editorial Policy Checklist.

Statistics

For all statistical analyses, confirm that the following items are present in the figure legend, table legend, main text, or Methods section.

n/a	Confirmed	
☐	☒	The exact sample size (*n*) for each experimental group/condition, given as a discrete number and unit of measurement
☐	☒	A statement on whether measurements were taken from distinct samples or whether the same sample was measured repeatedly
☐	☒	The statistical test(s) used AND whether they are one- or two-sided<br>*Only common tests should be described solely by name; describe more complex techniques in the Methods section.*
☒	☐	A description of all covariates tested
☐	☒	A description of any assumptions or corrections, such as tests of normality and adjustment for multiple comparisons
☐	☒	A full description of the statistical parameters including central tendency (e.g. means) or other basic estimates (e.g. regression coefficient) AND variation (e.g. standard deviation) or associated estimates of uncertainty (e.g. confidence intervals)
☐	☒	For null hypothesis testing, the test statistic (e.g. *F*, *t*, *r*) with confidence intervals, effect sizes, degrees of freedom and *P* value noted<br>*Give P values as exact values whenever suitable.*
☒	☐	For Bayesian analysis, information on the choice of priors and Markov chain Monte Carlo settings
☒	☐	For hierarchical and complex designs, identification of the appropriate level for tests and full reporting of outcomes
☒	☐	Estimates of effect sizes (e.g. Cohen's *d*, Pearson's *r*), indicating how they were calculated

Our web collection on statistics for biologists contains articles on many of the points above.

Software and code

Policy information about availability of computer code

Data collection	Whole pathology slide scanning was carried out using a Vectra 3 automated quantitative pathology imaging system (PerkinElmer), viewed and annotated by the ImageScope software (Leica, 12.3.3). Flow cytometry samples were analyzed using a BD FACSCanto II device (BD Biosciences). Wound Healing Assay were visualized and photographed under an inverted microscope (40 × objective) (Leica, Solms, Germany). Mass spectra were acquired using a Voyager DE-STR MALDI–time-of-flight mass spectrometer (software version 5.1; Applied Biosystems) in reflector mode. Quantitative PCR data were obtained with the Bio-Rad CFX96 Real-Time System.
Data analysis	Quantification of tissue staining were performed using the inForm software program (version 2.2; PerkinElmer). Flow cytometry data were analyzed using the FlowJo software program (version 10.0.8; Tree Star). Human Plk3 was modelled by Rosetta structural prediction server (https://robetta.bakerlab.org/) using comparative modeling. The missing residues are modelled in based on the secondary structure prediction in the Rosetta program. The raw data of mass spectrometry were processed using Thermo ScientificTM Proteome DiscovererTM software version 1.4. Spectra were searched against the Uniport-Homo sapiens database using the Mascot search engine (v 1.30, Matrix Science). Fragment match tolerance used for search: 0.8 Da; Fragments used for search: b; b-H_2O; b-NH_3; y; y-H_2O; y-NH_3. Carbamidomethylation on cysteine residues was used as fixed modification; phosphorylation of serine and threonine was set as variable modifications. The quantification of Western Blot signal was analyzed by Image J software. Statistical analysis was performed using the Graph Pad Prism 10.0.3 software program (GraphPad Software).

For manuscripts utilizing custom algorithms or software that are central to the research but not yet described in published literature, software must be made available to editors and reviewers. We strongly encourage code deposition in a community repository (e.g. GitHub). See the Nature Portfolio guidelines for submitting code & software for further information.

Data

Policy information about availability of data

All manuscripts must include a data availability statement. This statement should provide the following information, where applicable:

- Accession codes, unique identifiers, or web links for publicly available datasets
- A description of any restrictions on data availability
- For clinical datasets or third party data, please ensure that the statement adheres to our policy

Authors can confirm that all relevant data are included in the paper and/or its Supplementary Information files. Source data are provided with this paper. Plk3 expression in the Segara and Logsdon cancer microarray datasets are obtained from Oncomine (https://www.oncomine.com/). TCGA gene expression data were obtained from The Cancer Genome Atlas data portal (https://tcgadata.nci.nih.gov/tcga/dataAccessMatrix.htm) (Nature. 2008 Oct 23; 455(7216): 1061–1068). The Mass-spec data generated for this study are deposited in PRIDE under accession PXD042993 [https://www.ebi.ac.uk/pride/archive/projects/PXD042993]. The following PDB were used for modeling Plk3 structure: The Plk1 ortholog from Zebrafish (PDB:4j7b), Plk3 kinase domain (PDB: 4b61), and Plk2 Polo-box domain (PDB: 4rs6).

Research involving human participants, their data, or biological material

Policy information about studies with human participants or human data. See also policy information about sex, gender (identity/presentation), and sexual orientation and race, ethnicity and racism.

Reporting on sex and gender	N/A
Reporting on race, ethnicity, or other socially relevant groupings	N/A
Population characteristics	Our research did not involve human subjects, but used human samples obtained from Pathologist Dr. Huamin Wang at the University of Texas MD Anderson Cancer Center. All primary PDAC and paired adjacent normal pancreatic tissue specimens were collected within 1 h after surgery under a protocol approved by the MD Anderson Institutional Review Board, and written informed consent was obtained from all patients at the time of enrollment. They were shared with us as de-identified specimens.
Recruitment	We did not recruit any patients in this study.
Ethics oversight	The protocol was approved by Institutional Review Board at the University of Texas MD Anderson Cancer Center.

Note that full information on the approval of the study protocol must also be provided in the manuscript.

Field-specific reporting

Please select the one below that is the best fit for your research. If you are not sure, read the appropriate sections before making your selection.

☒ Life sciences ☐ Behavioural & social sciences ☐ Ecological, evolutionary & environmental sciences

For a reference copy of the document with all sections, see nature.com/documents/nr-reporting-summary-flat.pdf

Life sciences study design

All studies must disclose on these points even when the disclosure is negative.

Sample size	The number of mice used in each experimental group of genetic mice or orthotopic mouse models was based on prior experience with metastatic animal models (Cancer Cell. 2012 Jan 17;21(1):105-120. doi: 10.1016/j.ccr.2011.12.006; Nature Communications. 2016 Feb 24; 8: 14437. DOI: 10.1038/ncomms14437). For orthotopic mouse model experiments, sample size of n=5-7 was typical of experiments and was used in the study. For in vitro experiments, we used sample sizes containing 3 or more biological replicates which can provide adequate statistical power with good reproducibility in biochemical analysis. All sample sizes were listed in the corresponding figure legend or figures.
Data exclusions	No data was excluded from the study.
Replication	Except for the animal studies (one time) and tissue microarray analysis (one time), each experiment was repeated at least three times with similar results.
Randomization	For in vitro experiments, cells were randomly allocated into control and experimental groups. For in vivo experiments, age-matched mice were randomized into control and experimental groups prior to tumor size measurement. The micrographs, Flow cytometry analysis are in a random order.
Blinding	For human tissue microarray, IHC staining was scored semiquantitatively in a blinded fashion by two gastrointestinal pathologists. For cell-based experiments, blinding was not performed, because the investigator had to know the groups to load the samples or perform the assay. Blinding was not performed in orthotopic mouse model experiments, because the investigator needed to know the cell injection groups in order to perform the study.

Reporting for specific materials, systems and methods

We require information from authors about some types of materials, experimental systems and methods used in many studies. Here, indicate whether each material, system or method listed is relevant to your study. If you are not sure if a list item applies to your research, read the appropriate section before selecting a response.

Materials & experimental systems

n/a	Involved in the study
☐	☒ Antibodies
☐	☒ Eukaryotic cell lines
☒	☐ Palaeontology and archaeology
☒	☐ Animals and other organisms
☒	☐ Clinical data
☒	☐ Dual use research of concern
☒	☐ Plants

Methods

n/a	Involved in the study
☒	☐ ChIP-seq
☐	☒ Flow cytometry
☒	☐ MRI-based neuroimaging

Antibodies

Antibodies used

-Antibodies used for immunoblotting:
Rabbit monoclonal anti-Plk3 (for immunoblotting C-terminal Plk3), Cell Signaling Technology 4896; RRID:AB_10544409
Rabbit monoclonal anti-Plk1 (for immunoblotting C-terminal Plk1), Cell Signaling Technology 4513; RRID: AB_2167409
Rabbit polyclonal anti-cleaved PARP, Cell Signaling Technology 9542; RRID:AB_2160739
Rabbit monoclonal anti-cleaved caspase 3, Cell Signaling Technology 9664; RRID:AB_2070042
Rabbit monoclonal anti-c-Fos, Cell Signaling Technology 2250; RRID:AB_2247211
Rabbit monoclonal anti-V5 Tag ,Cell Signaling Technology 13202; RRID:AB_2687461
Mouse monoclonal anti-Myc Tag, Cell Signaling Technology 2276; RRID:AB_331783
Rabbit monoclonal anti-PI3 Kinase p110a, Cell Signaling Technology 4249; RRID:AB_2165248
Rabbit monoclonal anti-HA Tag, Cell Signaling Technology 3724; RRID:AB_1549585
Rabbit polyclonal anti-Lamin A/C, Cell Signaling Technology 2032; RRID:AB_2136278
Rabbit monoclonal anti-c-Jun, Cell Signaling Technology 9165; RRID:AB_2130165
Rabbit polyclonal anti-Histone H3, Cell Signaling Technology 9715; RRID:AB_331563
Rabbit polyclonal anti-Phospho-Histone H3 (Ser10), Cell Signaling Technology 9701, RRID:AB_331535
Rabbit polyclonal anti-Cyclin B1, Cell Signaling Technology 4138, RRID:AB_2072132
Mouse monoclonal anti-Cyclin D1, Cell Signaling Technology 2926, RRID:AB_2070400
Mouse monoclonal anti-Cyclin E1, Cell Signaling Technology 4129; RRID:AB_2071200
Goat polyclonal anti-Plk3 (for immunoblotting N-terminal Plk3), LSBio LS-B10518
Rabbit polyclonal anti-Plk3 (for immunoblotting mouse N-terminal Plk3), LSBio LS-C383512
Rabbit polyclonal anti-Plk1 (for immunoblotting N-terminal Plk1), Abcam ab70695; RRID:AB_1269814
Rabbit monoclonal anti-Thiophosphate ester, Abcam ab92570, RRID:AB_10562142
Mouse monoclonal anti-Flag(R) M2, Sigma A8592; RRID:AB_439702
Mouse monoclonal anti-b-actin, Sigma A5316;RRID:AB_476743
Rabbit polyclonal anti-c-Fos, Santa Cruz Biotechnology sc-7202; RRID:AB_2106765
Mouse monoclonal anti-Nardilysin, Santa Cruz Biotechnology sc-137199; RRID:AB_2154523
Goat polyclonal anti-Centaurin alpha 1, Novus Biologicals NB300-907; RRID:AB_2273580
Mouse monoclonal anti-Vinculin, Millipore 05-386; RRID:AB_11212640
Mouse polyclonal anti-p-cFosT164A, This study N/A

-Antibodies used for IHC:
Rabbit polyclonal anti-cleaved caspase 3, Cell Signaling Technology 9661; RRID:AB_2341188
Rabbit polyclonal anti-Plk3, Novus Biologicals NBP2-32530
Rabbit monoclonal anti-Ki-67, ThermoFisher Scientific RM-9106; RRID:AB_2335745

-Antibodies used for flow cytometry:
FITC Mouse Anti- BrdU, BD Biosciences 556028, RRID:AB_396304
Annexin V Recom APC antibody, BD Biosciences 550474; RRID:AB_2868885

-Antibodies used for Immunofluorescence:
Rabbit polyclonal anti-Plk3, Abcam ab123695; RRID:AB_10949660
Mouse monoclonal anti-c-Fos, Santa Cruz Biotechnology sc-271243; RRID:AB_10610067
Donkey Anti-Rabbit IgG (H+L), Life Technologies A21206; RRID: AB_2535792
Donkey Anti-Rabbit IgG (H+L), Life Technologies A21207; RRID:AB_141637

Validation

Pre-validated antibodies were purchased from reputable sources and are stated to be tested by the manufacturer for species reactivity to human or mouse. The statements and validation data for each primary antibody for the species and application are also

available on the manufacturers website. In addition antibodies were monitored in house to detect protein of the reported size by western blot in over expression and/or knock down experiments.
-Antibodies used for immunoblotting:
Rabbit monoclonal anti-Plk3 (for immunoblotting C-terminal Plk3), Cell Signaling Technology 4896; RRID:AB_10544409, https://www.cellsignal.com/products/primary-antibodies/plk3-d14f12-rabbit-mab/4896
Rabbit monoclonal anti-Plk1 (for immunoblotting C-terminal Plk1), Cell Signaling Technology 4513; RRID: AB_2167409, https://www.cellsignal.com/products/primary-antibodies/plk1-208g4-rabbit-mab/4513
Rabbit polyclonal anti-cleaved PARP, Cell Signaling Technology 9542; RRID:AB_2160739, https://www.cellsignal.com/products/primary-antibodies/parp-antibody/9542
Rabbit monoclonal anti-cleaved caspase 3, Cell Signaling Technology 9664; RRID:AB_2070042, https://www.cellsignal.com/products/primary-antibodies/cleaved-caspase-3-asp175-5a1e-rabbit-mab/9664
Rabbit monoclonal anti-c-Fos, Cell Signaling Technology 2250; RRID:AB_2247211, https://www.cellsignal.com/products/primary-antibodies/c-fos-9f6-rabbit-mab/2250
Rabbit monoclonal anti-V5 Tag, Cell Signaling Technology 13202; RRID:AB_2687461, https://www.cellsignal.com/products/primary-antibodies/v5-tag-d3h8q-rabbit-mab/13202
Mouse monoclonal anti-Myc Tag, Cell Signaling Technology 2276; RRID:AB_331783, https://www.cellsignal.com/products/primary-antibodies/myc-tag-9b11-mouse-mab/2276
Rabbit monoclonal anti-PI3 Kinase p110a Cell Signaling Technology 4249; RRID:AB_2165248, https://www.cellsignal.com/products/primary-antibodies/pi3-kinase-p110a-c73f8-rabbit-mab/4249
Rabbit monoclonal anti-HA Tag, Cell Signaling Technology 3724; RRID:AB_1549585, https://www.cellsignal.com/products/primary-antibodies/ha-tag-c29f4-rabbit-mab/3724
Rabbit polyclonal anti-Lamin A/C, Cell Signaling Technology 2032; RRID:AB_2136278, https://www.cellsignal.com/products/primary-antibodies/lamin-a-c-antibody/2032
Rabbit monoclonal anti-c-Jun, Cell Signaling Technology 9165; RRID:AB_2130165, https://www.cellsignal.com/products/primary-antibodies/c-jun-60a8-rabbit-mab/9165
Rabbit polyclonal anti-Histone H3, Cell Signaling Technology 9715; RRID:AB_331563, https://www.cellsignal.com/products/primary-antibodies/histone-h3-antibody/9715
Rabbit polyclonal anti-Phospho-Histone H3 (Ser10), Cell Signaling Technology 9701, RRID:AB_331535, https://www.cellsignal.com/products/primary-antibodies/phospho-histone-h3-ser10-antibody/9701
Rabbit polyclonal anti-Cyclin B1, Cell Signaling Technology 4138, RRID:AB_2072132, https://www.cellsignal.com/products/primary-antibodies/cyclin-b1-antibody/4138
Mouse monoclonal anti-Cyclin D1, Cell Signaling Technology 2926, RRID:AB_2070400, https://www.cellsignal.com/product/productDetail.jsp?productId=2926
Mouse monoclonal anti-Cyclin E1, Cell Signaling Technology 4129; RRID:AB_2071200, https://www.cellsignal.com/products/primary-antibodies/cyclin-e1-he12-mouse-mab/4129?_=1662153768319&Ntt=4129&tahead=true
Goat polyclonal anti-Plk3 (for immunoblotting N-terminal Plk3), LSBio LS-B10518, https://www.lsbio.com/antibodies/ihc-plus-plk3-antibody-aa320-333-ihc-wb-western-ls-b10518/295183
Rabbit polyclonal anti-Plk3 (for immunoblotting mouse N-terminal Plk3), LSBio LS-C383512, https://www.lsbio.com/antibodies/plk3-antibody-200-280-aa-internal-elisa-wb-western-ls-c383512/395613
Rabbit polyclonal anti-Plk1 (for immunoblotting N-terminal Plk1), Abcam ab70695; https://www.abcam.com/plk1-antibody-ab70695.htmlRRID:AB_1269814
Rabbit monoclonal anti-Thiophosphate ester, Abcam ab92570, RRID:AB_10562142, https://www.abcam.com/thiophosphate-ester-antibody-51-8-ab92570.html
Mouse monoclonal anti-Flag(R) M2, Sigma A8592; RRID:AB_439702, https://www.sigmaaldrich.com/US/en/product/sigma/a8592?gclid=Cj0KCQjw08aYBhDlARIsAA_gb0c9MvdRn2vAz9yg7RuBc_ytGqcm30PrN2Ywnzu2gqtckuDUxlbpNsMaAuYEEALw_wcB
Mouse monoclonal anti-b-actin, Sigma A5316; RRID:AB_476743, https://www.sigmaaldrich.com/US/en/search/a5316?focus=products&page=1&perpage=30&sort=relevance&term=a5316&type=product
Rabbit polyclonal anti-c-Fos, Santa Cruz Biotechnology sc-7202; RRID:AB_2106765, https://www.scbt.com/p/c-fos-antibody-h-125
Mouse monoclonal anti-Nardilysin, Santa Cruz Biotechnology sc-137199; RRID:AB_2154523, https://www.scbt.com/p/nardilysin-antibody-a-6?requestFrom=search
Goat polyclonal anti-Centaurin alpha 1, Novus Biologicals NB300-907; RRID:AB_2273580, https://www.novusbio.com/products/centaurin-alpha-1-antibody_nb300-907
Mouse monoclonal anti-Vinculin, Millipore 05-386; RRID:AB_11212640, https://www.emdmillipore.com/US/en/product/Anti-Vinculin-Antibody-clone-V284,MM_NF-05-386

-Antibodies used for IHC:
Rabbit polyclonal anti-cleaved caspase 3, Cell Signaling Technology 9661; RRID:AB_2341188, https://www.cellsignal.com/products/primary-antibodies/cleaved-caspase-3-asp175-antibody/9661
Rabbit polyclonal anti-Plk3, Novus Biologicals NBP2-32530, https://www.novusbio.com/products/plk3-antibody_nbp2-32530
Rabbit monoclonal anti-Ki-67, ThermoFisher Scientific RM-9106; RRID:AB_2335745, https://tools.thermofisher.com/content/sfs/brochures/D12536~.pdf

-Antibodies used for flow cytometry:
FITC Mouse Anti-BrdU, BD Biosciences 556028, RRID:AB_396304, https://www.bdbiosciences.com/en-us/products/reagents/flow-cytometry-reagents/research-reagents/panels-multicolor-cocktails-ruo/fitc-mouse-anti-brdu-set.556028
Annexin V Recom APC antibody, BD Biosciences 550474; RRID:AB_2868885, https://www.bdbiosciences.com/en-us/search-results?searchKey=550474

-Antibodies used for Immunofluorescence:
Rabbit polyclonal anti-Plk3, Abcam ab123695; RRID:AB_10949660, https://www.abcam.com/plk3-antibody-ab123695.html
Mouse monoclonal anti-c-Fos, Santa Cruz Biotechnology sc-271243; RRID:AB_10610067, https://www.scbt.com/p/c-fos-antibody-c-10

Donkey Anti-Rabbit IgG (H+L), Life Technologies A21206; RRID: AB_2535792, https://www.thermofisher.com/antibody/product/Donkey-anti-Rabbit-IgG-H-L-Highly-Cross-Adsorbed-Secondary-Antibody-Polyclonal/A-21206
Donkey Anti-Rabbit IgG (H+L), Life Technologies A21207; RRID:AB_141637, https://www.thermofisher.com/antibody/product/Donkey-anti-Rabbit-IgG-H-L-Highly-Cross-Adsorbed-Secondary-Antibody-Polyclonal/A-21207

Mouse polyclonal anti-p-cFosT164A was generated in house. cFos T164 specific hot peptide C-RELTD-(pT164)-LQAETDQLEDE and one cold peptide C-RELTD-(T164)-LQAETDQLEDE were synthesized. 20 mice were immunized using pT164 peptide once every two weeks for five rounds of injections. Antisera was collected and tittered by ELISA. The specificity of these T164-p antisera was individually examined with dot blotting/peptide blocking experiment. The T164-p antibody used in our study was selected from candidate antisera that specifically bind with the hot peptide (T164-p) while not binding with the cold peptide (T164) and exhibit strong signal.

Eukaryotic cell lines

Policy information about cell lines and Sex and Gender in Research

Cell line source(s)	Human pancreatic cancer cell lines MIA PaCa-2, PANC-1, BxPC-3, AsPC-1, Capan-1, and Colo357, the human colorectal carcinoma cell line HCT116, and the human embryonic kidney cell line HEK293T were obtained from the American Type Culture Collection (ATCC). PDAC cell lines MDA-PATC43, 50, 53, 66, 69, 102, 107, 108, 124, 148, 148LM, 148LM2, 153, 153LM, 216, and 219B were provided by Dr. Jason B. Fleming (MD Anderson). Tumorigenic HPDE/KrasG12V/Her2/shp16shp14/shSmad4 cells were established via stable expression of mutant Kras, Her2, p16, p14 shRNA, and Smad4 shRNA in HPDE cells. KIC and KPC mouse-derived cell lines were generated independently in house from pancreatic tumors harvested from p48-cre;KrasLSL-G12D;INK4aF/F and p48-cre;KrasLSL-G12D;p53LSL-H172R mice. PTEN+/+ and PTEN-/- MEFs were gifts from Dr. Min Sup Song (MD Anderson). Normal mouse pancreatic epithelial cell lines, P72Plk3-WT, Plk3-heterozygous, and Plk3-KO primary MEFs were generated in house.
Authentication	Cell lines are authenticated by the MD Anderson Cancer Center Characterized Cell Line Core Facility by STR profiling.
Mycoplasma contamination	All cell lines were tested and found to be free of mycoplasma contamination.
Commonly misidentified lines (See ICLAC register)	No commonly misidentified cell lines were used.

Flow Cytometry

Plots

Confirm that:

- [x] The axis labels state the marker and fluorochrome used (e.g. CD4-FITC).
- [x] The axis scales are clearly visible. Include numbers along axes only for bottom left plot of group (a 'group' is an analysis of identical markers).
- [x] All plots are contour plots with outliers or pseudocolor plots.
- [x] A numerical value for number of cells or percentage (with statistics) is provided.

Methodology

Sample preparation	Cells were obtained from cell culture plates by trypsinization, blocking and staining according to the manufacturer's protocol. Cell surface staining was performed at 4C for 30 min.
Instrument	BD FACSCanto II cytometer (BD Biosciences)
Software	FlowJo software program (version 10.0.8; Tree Star)
Cell population abundance	At least 5,000 cells were analyzed for each sample.
Gating strategy	The first gating step was to plot cells on the basis of the cells' forward scatter (FSC-A) and side scatter (SSC-A) properties. Second, single cells were identified by plotting their FSC-height (FSC-H) by FSC-area (FSC-A). Third, fluorophore positive cells were identified by the respective channels.

- [x] Tick this box to confirm that a figure exemplifying the gating strategy is provided in the Supplementary Information.
